# Effect of ethnomedicinal plants used in folklore medicine in Jordan as antibiotic resistant inhibitors on *Escherichia coli*

**DOI:** 10.1186/1472-6882-10-9

**Published:** 2010-02-28

**Authors:** Rula M Darwish, Talal A Aburjai

**Affiliations:** 1 Department of Pharmaceutics and Pharmaceutical Technology Faculty of Pharmacy, University of Jordan, Amman, Jordan; 2 Department of Pharmaceutical Sciences, Faculty of Pharmacy, University of Jordan, Amman, Jordan

## Abstract

**Background:**

*Escherichia coli *occurs naturally in the human gut; however, certain strains that can cause infections, are becoming resistant to antibiotics. Multidrug-resistant *E. coli *that produce extended-spectrum β lactamases (ESBLs), such as the CTX-M enzymes, have emerged within the community setting as an important cause of urinary tract infections (UTIs) and bloodstream infections may be associated with these community-onsets. This is the first report testing the antibiotic resistance-modifying activity of nineteen Jordanian plants against multidrug-resistant *E. coli*.

**Methods:**

The susceptibility of bacterial isolates to antibiotics was tested by determining their minimum inhibitory concentrations (MICs) using a broth microdilution method. Nineteen Jordanian plant extracts (*Capparis spinosa *L., *Artemisia herba-alba Asso, Echinops polyceras *Boiss., *Gundelia tournefortii *L, *Varthemia iphionoides *Boiss. & Blanche, *Eruca sativa Mill*., *Euphorbia macroclada *L., *Hypericum trequetrifolium *Turra, *Achillea santolina *L., *Mentha longifolia *Host, *Origanum syriacum *L., *Phlomis brachydo*(Boiss.) Zohary, *Teucrium polium *L., *Anagyris foetida *L., *Trigonella foenum-graecum *L., *Thea sinensis *L., *Hibiscus sabdariffa *L., *Lepidium sativum *L., *Pimpinella anisum *L.) were combined with antibiotics, from different classes, and the inhibitory effect of the combinations was estimated.

**Results:**

Methanolic extracts of the plant materials enhanced the inhibitory effects of chloramphenicol, neomycin, doxycycline, cephalexin and nalidixic acid against both the standard strain and to a lesser extent the resistant strain of *E. coli*. Two edible plant extracts (*Gundelia tournefortii L*. and *Pimpinella anisum L*.) generally enhanced activity against resistant strain. Some of the plant extracts like *Origanum syriacum *L.(Labiateae), *Trigonella foenum- graecum *L.(Leguminosae), *Euphorbia macroclada *(Euphorbiaceae) and *Hibiscus sabdariffa *(Malvaceae) did not enhance the activity of amoxicillin against both standard and resistant *E. coli*. On the other hand combinations of amoxicillin with other plant extracts used showed variable effect between standard and resistant strains. Plant extracts like *Anagyris foetida *(Leguminosae) and *Lepidium sativum *(Umbelliferae) reduced the activity of amoxicillin against the standard strain but enhanced the activity against resistant strains. Three edible plants; Gundelia *tournefortii *L. (Compositae) *Eruca sativa *Mill. (Cruciferae), and *Origanum syriacum *L. (Labiateae), enhanced activity of clarithromycin against the resistant *E. coli *strain.

**Conclusion:**

This study probably suggests possibility of concurrent use of these antibiotics and plant extracts in treating infections caused by *E. coli *or at least the concomitant administration may not impair the antimicrobial activity of these antibiotics.

## Background

*E. coli *occurs naturally in the human gut; however, certain strains that can lead to infections are becoming resistant to antibiotics. From the late 1990s, multidrug-resistant *Enterobacteriaceae *(mostly *Escherichia coli*) that produce extended-spectrum β lactamases (ESBLs), such as the CTX-M enzymes, have emerged within the community setting as an important cause of urinary tract infections (UTIs) [1]. Recent reports have also described ESBL-producing *E. coli *as a cause of bloodstream infections associated with these community-onsets of UTI [2]. Such development of drug resistance in human pathogens against commonly used antibiotics has necessitated a search for new antimicrobial substances, chemotherapeutic agents, and agrochemicals that combine antimicrobial efficacy with low toxicity, and minor environmental impact.

Natural products offer an untold diversity of chemical structures. These natural compounds often serve as lead molecules whose activities can be enhanced by manipulation through combinations with chemicals and by synthetic chemistry [3,4].

An important source of natural products is plants which are rich in a wide variety of secondary metabolites, such as tannins, terpenoids, alkaloids, and flavonoids. These metabolites have been found in vitro to have antimicrobial properties [5-14]. Interest in medicinal plants has increased in recent years. This interest has lead to the discovery of new biologically-active molecules by the pharmaceutical industry and the adoption of crude extracts of plants for self-medication by the general public [3,4].

Many plants have been evaluated not only for their inherent antimicrobial activity, but also for their action as a resistance-modifying agent [15-18]. The enhancement of antibiotic activity or the reversal of antibiotic resistance by natural or synthetic non-conventional antibiotics has lead to the classification of these compounds as modifiers of antibiotic activity.

In this study we screened nineteen Jordanian plants, known to have antimicrobial activity in folk medicine [19-23], for their possible effect as modifiers of antibiotic activity against bacteria. Some of them are edible and considered safe. In general, these plants are used in folk medicine in the treatment of skin diseases, gastrointestinal tract diseases and respiratory problems. The plants used in this study and their properties are listed in Table 1. Relative few studies have been carried out to evaluate the antimicrobial properties of these plants. Two strains of *E. coli *were used, a resistant strain, which was isolated from a local hospitalized patient, and a standard laboratory strain from the ATCC culture collection.

**Table 1:** Uses and properties of ethnomedicinal plants used in this study.

	Family Name	Scientific Name (voucher specimen)	% yield	Part used	Claimed Usage	
1	Capparidaceae	*Capparis spinosa *L. (Abbadi 99-20)	6	Roots	Rheumatic pain Purgative and anthelmentic	Internally: decoction Externally: paste of the root bark of the plant is mixed with dough and applied on the site of pain for 10-20 min
2	Compositae	*Artemisia herba-alba Asso*. (Abbadi 00-8)	4.5	Foliage	Antidiabetic, Antispasmodic, pectoral, antiarthritis	Infusion of 30 g in 1 L of water
3	Compositae	*Echinops polyceras *Boiss. (Al-abd. 99-3)	9.1	Whole plant	Wounds and warts	pulverized powder of the plant is applied directly on affected area
4	Compositae	*Gundelia tournefortii *L (Abbadi 00-24)	6.7	Whole plant	Edible like artichoke, antioxidant, treatment of vitiligo, diuretic	Internally: Cooking, decoction Externally: a paste from the plant prepared with Vaseline and applied
5	Compositae	*Varthemia iphionoides *Boiss. & Blanche (VaI-M99)	8.8	Leaves and Stems	Women sterility, female fertilization, eye infection, antispasmodic Anti-inflammatory, diabetes. Women delivery	Infusion, Vapor, Lotion, Vapors after burning with Harmal
6	Cruciferae	*Eruca sativa Mill*. (ES-M99)	11.2	Fruits	Aphrodisiac Antispasmodic and for renal colic	Decoction.
7	Euphorbiaceae	*Euphorbia macroclada *L. (Al-abd. 98-11)	0.8	Latex	Urticaria, warts	One drop of stem sap is applied directly to affected areas only
8	Euphorbiaceae	*Euphorbia macroclada *L. (Al-abd. 98-1)	6.6	whole plant	Urticaria, warts	Decoction or pulverized powder of the plant is applied directly on affected area
9	Gittiferae	*Hypericum trequetrifolium *Turra (Abbadi 99-23)	5.5	Arial parts	Toxic Antidepressant in cases of mania	Not used medicinally
10	Labiateae	*Achillea santolina *L. (AS-M99)	7.9	Aerial Parts	Carminative, Depurative, Stomachaches, antispasmodic and diabetes	Infusion and Decoction are prepared in water and taken orally 3 times daily
11	Labiateae	*Mentha longifolia *Host (ML-99)	10.6	Leaves	Constipation, fever, common cold, general weakness	Infusion of the plant is made with water or tea.
12	Labiateae	*Origanum syriacum *L. (Majorana syriaca (L.) Raf. (Abbadi 00-19)	9.6	Leaves	Carminative, pectoral, antitussive, aperative, antistomach ache, Carminative.	Infusion
13	Labiateae	*Phlomis brachydon* (Boiss.) Zohary (Al-Abd. 99-4)	3.6	Whole plant	Stomach and intestine pain	Decoction.
14	Labiateae	Teucrium polium L. (Abbadi 99-5)	11.9	Aerial parts	Spasm, flatulence, diabetes and kidney stones	Infusion is prepared and taken orally three times a day.
15	Leguminosae	*Anagyris foetida *L. (Al-abd. 99-2)	8.6	Leaves & fruits	laxative, pectoral, purgative, vermifuge	Decoction.
16	Leguminosae	Trigonella foenum-graecum L. (TF-M99)	6.3	Seeds	Diabetes, sexual impotence, intestinal pain, infant abdominal pain, skin diseases	A decoction is prepared from the seeds and taken orally 3 times daily. For infants a poultice is mad from the seed and fixed in the site of pain. Externally the decoction is used as a lotion.
17	Theaceae	*Thea sinensis *L. (TS-m99)	5.6	leaves	Drink, externally anti-inflammatory	Decoction.
18	Malvaceae	*Hibiscus sabdariffa *L. (Abbadi 00-180)	6.4	Calyx	Drink, antihypertensive	Decoction.
19	Umbelliferae	*Lepidium sativum *L. (LS-M99)	3.8	Seeds. Fresh plant	General tonic	Infusion. The fresh plant is added to salad or eaten as green vegetable.
20	Umbelliferae	*Pimpinella anisum *L. (PA-M99)	9.9	Fruit	Antiflatulance and antispasmodic	Infusion.

## Methods

### Plant material

Plants were either collected from the field or purchased from the local market (Table 1). The taxonomic identity of the plants was confirmed by comparing collected voucher specimens with those of known identity which are located in the Herbarium of the Dept. of Biological Science, Faculty of Science, University of Jordan in Amman.

### Preparation of plant extracts

Air dried and finely powdered plant materials were extracted in a Soxhlet with two liters of methanol for 4 hrs, except for *Capparis spinosa*, which was extracted for 10 hrs. Methanol is a semi polar solvent and is used in extracting polar and apolar compounds simultaneously.

Solvents were then evaporated under reduced pressure and the extracts were conserved in tightly sealed glass vials. *Euphorbia macroclada *latex was obtained by cutting and squeezing the stem of the plant and examined directly.

### Determination of antimicrobial activity

#### Microorganisms

A resistant strain of *Escherichia coli *was isolated from hospitalized patients from the Jordan University Hospital and its identity confirmed by biochemical tests. A standard laboratory strain of *E. coli *ATCC 8739 was used as control.

#### Preparation of inoculum

Stock cultures were maintained at 4°C on slopes of nutrient agar. Cultures for experiments were prepared by transferring a sample from the stock cultures into Mueller-Hinton broth (MHB) and incubating without agitation for 24 hrs at 37°C. The cultures were diluted with fresh Mueller-Hinton broth to achieve optical densities corresponding to 2.0 × 10^6 ^colony forming units (CFU/ml).

#### Antibiotics

Antibiotics used in this study were amoxicillin, (Merck), chloramphenicol, (Fluka), neomycin, (Hikma Pharmaceutical Manufacture Co.), cephalexin, clarithromycin, doxycycline, (Arab Pharmaceutical Manufacture Co) and Nalidixic acid (Fluka).

### Minimum inhibitory concentration (MIC) determination for antibiotics

The MIC of the antibiotics was tested by the NCCLS broth microdilution reference method [24] with some modification. MIC tests were performed in 96 flat bottom microtiter plates (TPP, Switzerland). Each test well was filled with 100 μl nutrient broth. A sample (100 μl) of the antibiotic stock solution was added to the first test well and mixed. A series of dilutions was then prepared across the plate using a micropipette. The concentration ranges used to determine MICs were: Amoxycillin 0.12-32 μg/ml, Chlormphenicol 0.5- 30 μg/ml, Nalidixic acid 0.12-16 μg/ml, Cephalexin 0.5-42 μg/ml, Neomycin 2-256 μg/ml, Doxycycline 0.5-128 μg/ml and Clarithromycin 0.5-160 μg/ml. A 10 μl aliquot of the standard laboratory strain of *E. coli *ATCC 8739 was used to inoculate each microtiter plate well to achieve a final inoculum size of 5 × 10^5 ^CFU/ml.

Positive growth controls (well with overnight culture, nutrient broth and bacterial inoculum but without antibiotic) and negative controls (well with broth but without inoculum) were also prepared and incubated at 37° for 24 hrs.

Microbial growth in the test wells was detected as turbidity, visualized by naked eyes, relative to the negative and positive controls. MICs were calculated as follows:

Where

C n: Concentration at well number n, where no turbidity was observed.

C (n + 1): Concentration at well number (n + 1), where turbidity was observed.

MIC determination was carried out in triplicate (in same 96-well plate) and repeated twice for each bacteria and each tested agent. MICs values are shown in Table 2.

**Table 2:** Minimum inhibitory concentrations of the antibiotics used in the study against standard laboratory strain of *Escherichia coli *ATCC 8739

Antimicrobial agent	MIC (μg/ml)
Amoxicillin	16

Chloramphenicol	25

Neomycin	64

Doxycycline	32

Clarithromycin	150

Cephalexin	32

Nalidixic acid	8

### Modification of antibiotic activity by plant extracts

Antibiotics were added to 18.5 ml molten nutrient agar to give half their MIC concentrations (Table 2). Dried plant extracts were dissolved in absolute ethanol to give a stock solution of 8 mg/ml. To determine the effect of the plant extract on the activity of the antibiotics, 0.5 ml of the ethanolic solution of the plant extract and 1 ml of the bacterial suspension was added to the nutrient agar containing the antibiotic to give an inoculum size of 5 × 10 ^3 ^CFU/ml cells and a final concentration of 200 μg/ml of the plant extract in the nutrient gar. The medium was mixed thoroughly, poured in a plate and then incubated at 37°C for 24 hrs. The number of colonies on each plate (N) was determined. At the same time, for each combination control counts (N_0_) which were determined by adding an inoculum size of 5 × 10^3 ^CFU/ml to molten nutrient agar containing 0.5 ml of ethanol and incubating at 37°C for 24 hrs. The percentage growth was then calculated by reference to the control count (considered as 100% growth) as follows

Where

N_0 _is the number of colonies on the control count of the blank

N is the colony count after exposure to combinations of the antibiotic and the plant extract

Control and test counts were determined twice for each bacterial strain and for each combination of antibiotic and plant extract.

The percentage growth was determined twice for each bacterial strain and for each combination of antibiotic and plant extract. The test count was always referred to the control (100% growth) count done at the same time. The average percentage is presented in Tables 3 &4.

**Table 3:** Effect of each plant extract combined with various antibiotics on growth of resistant *E. coli*.

			%Growth on Combination with antibiotic^**a **^(± SE)^**b**^							
	Family	Plant^**c**^	Blank^**d**^	Amo*	Chl*	Neo*	Doxy*	Clarith*	Ceph*	Nal*
1	Capparidaceae	*Capparis spinosa *L	100	68.5 ± 4.6	75.9 ± 5.0	70.2 ± 2.9	40.5 ± 6.6	100 ± 4.2	76.8 ± 9.1	59.5 ± 5.7
2	Compositae	*Artemisia herba-alba *Asso.	100	85.9 ± 3.5	77.8 ± 2.5	50.1 ± 4.5	30.7 ± 8.3	65.9 ± 3.5	68.9 ± 4.9	77.9 ± 7.1
3	Compositae	*Echinops polyceras *Boiss	100	75.5 ± 2.2	85.9 ± 4.7	85.2 ± 2.3	40.5 ± 6.4	70.9 ± 5.7	79.9 ± 4.8	60.9 ± 5.1
4	Compositae	*Gundelia tournefortii *L	100	50.9 ± 8.8	60.9 ± 2.8	75 ± 3.7	30.5 ± 4.6	52.5 ± 5.1	80.8 ± 8.4	89 ± 4.2
5	Compositae	*Varthemia iphionoides *Boiss & Blanche	100	49 ± 4.9	60.1 ± 6.3	70.5 ± 5.4	64.0 ± 8.4	85.5 ± 6.2	60.9 ± 2.0	70.9 ± 2.4
6	Cruciferae	*Eruca sativa *Mill.	100	70.9 ± 6.5	51.6 ± 8.6	88.9 ± 2.3	25.5 ± 7.5	70.5 ± 4.2	60.5 ± 3.2	84.6 ± 4.9
7	Euphorbiaceae	*Euphorbia macroclada *L. (latex)	100	100.2 ± 1.7	80.8 ± 6.4	90.1 ± 8.4	45.7 ± 5.9	80.5 ± 8.1	72.5 ± 7.6	99.7 ± 8.1
8	Euphorbiaceae	*Euphorbia macroclada *L. (plant)	100	120.1 ± 6.3	80.8 ± 2.5	68.9 ± 3.9	26.2 ± 8.3	65.9 ± 4.7	89.9 ± 7.6	103 ± 2.8
9	Gittiferae	*Hypericum androsaemum *L	100	77.9 ± 1.5	55.9 ± 2.6	75.8 ± 3.5	6.5 ± 7.4	89.9 ± 5.5	67.8 ± 8.4	92.0 ± 3.9
10	Labiateae	*Achillea santolina *L.	100	70.5 ± 3.6	75 ± 3.3	61 ± 2.6	25 ± 4.9	70.2 ± 2.5	65.4 ± 5.6	75 ± 4.6
11	Labiateae	*Mentha piperita *L.	100	75.1 ± 2.5	64.9 ± 4.5	97.8 ± 2.2	25.5 ± 1.9	100 ± 3.5	90.6 ± 7.3	84.9 ± 2.5
12	Labiateae	*Origanum syriacum *L.	100	100 ± 4.9	70.9 ± 7.5	70.2 ± 6.5	30.6 ± 7.3	60.5 ± 5.6	55.8 ± 4.5	85.9 ± 3.2
13	Labiateae	*Phlomis brachydon *(Boiss.) Zohary	100	90.6 ± 2.4	100 ± 5.2	100 ± 4.6	30 ± 3.9	87.5 ± 6.2	60.1 ± 7.3	70.9 ± 5.1
14	Labiateae	*Teucrium polium *L.	100	68.9 ± 5.8	77.9 ± 2.5	85.5 ± 3.8	40.5 ± 7.3	70.5 ± 4.9	80.5 ± 2.6	96.9 ± 3.8
15	Leguminosae	*Anagyris foetida *L	100	69 ± 5.4	80.9 ± 4.6	90.6 ± 3.6	50.5 ± 5.4	89.9 ± 2.6	89.9 ± 4.2	99.9 ± 3.9
16	Leguminosae	*Trigonella foenum- graecum *L.	100	100 ± 8.4	88.9 ± 5.9	100 ± 3.2	55.5 ± 2.6	100 ± 7.1	90.5 ± 7.6	92.7 ± 7.2
17	Theaceae	*Thea sinensis *L.	100	85.9 ± 4.8	50.8 ± 6.1	70.7 ± 5.1	15.0 ± 2.5	75.8 ± 3.9	90 ± 4.5	104 ± 2.5
18	Malvaceae	*Hibiscus sabdariffa *L	100	120 ± 4.1	82.8 ± 5.6	85.5 ± 5.5	40.5 ± 6.4	80.7 ± 5.0	89.9 ± 6.4	80.5 ± 8.4
19	Umbelliferae	*Lepidium sativum *L.	100	50.9 ± 1.9	67.9 ± 7.9	89.9 ± 6.2	40.5 ± 3.7	50.9 ± 6.1	70.7 ± 4.7	90.1 ± 2.6
20	Umbelliferae	*Pimpinella anisum *L.	100	90.6 ± 3.3	66.9 ± 4.3	77.6 ± 1.6	25.5 ± 5.2	60.5 ± 4.2	84.6 ± 8.3	89.9 ± 4.9

^a^Amoxicillin (Amo), Chloramphenicol (Chl), Neomycin(Neo), Doxycycline (Doxy), Clarithromycin (Clarith), Cephalexin (Ceph) and Nalidixic acid (Nal).

^b^SE standard error

^c^plant extracts concentrations was (200 μg/ml)

* Antibiotics concentrations were half the MICs (presented in Table 2)

^c ^Blank containing the solvent with the nutrient agar and the bacteria (allowed full growth of the microorganism (100%).

**Table 4:** Effect of each plant extract combined with various antibiotics on growth of standard *E. coli*.

			%Growth on Combination with antibiotic^**a **^(± SE)^**b**^							
	Family	Plant^**c**^	Blank^**d**^	Amo*	Chl*	Neo*	Doxy*	Clarith*	Ceph*	Nal*
1	Capparidaceae	*Capparis spinosa *L	100	95.5 ± 5.9	NG ^d^	NG ^d^	4.0 ± 3.6	93.0 ± 6.1	NG ^d^	NG ^d^
2	Compositae	*Artemisia herba-alba *Asso.	100	92.7 ± 7.2	1 ± 4.9	NG ^d^	NG ^d^	89.0 ± 4.1	NG ^d^	NG ^d^
3	Compositae	*Echinops polyceras *Boiss	100	104.6 ± 6.1	NG ^d^	1.1 ± 3.5	2.5 ± 3.8	80.7 ± 4.6	1.1 ± 4.1	NG ^d^
4	Compositae	*Gundelia tournefortii *L	100	70.8 ± 3.9	1.1 ± 8.7	NG ^d^	NG ^d^	90.9 ± 2.7	NG ^d^	1.1 ± 5.6
5	Compositae	*Varthemia iphionoides *Boiss & Blanche	100	85.5 ± 8.6	NG ^d^	1.1 ± 5.4	NG ^d^	103.4 ± 4.0	NG ^d^	NG ^d^
6	Cruciferae	*Eruca sativa *Mill.	100	85.5 ± 4.2	1 ± 5.6	1.1 ± 9.4	1.1 ± 8.1	100 ± 5.9	1.1 ± 1.9	1.5 ± 2.5
7	Euphorbiaceae	*Euphorbia macroclada *L. (latex)	100	110 ± 6.4	NG ^d^	1.5 ± 5.6	1.1 ± 6.9	100 ± 4.3	1.9 ± 3.4	1.5 ± 1.8
8	Euphorbiaceae	*Euphorbia macroclada *L. (plant)	100	100.7 ± 5.9	NG ^d^	NG ^d^	NG ^d^	80.5 ± 7.3	NG ^d^	2.2 ± 3.8
9	Gittiferae	*Hypericum androsaemum *L	100	90.7 ± 6.6	NG ^d^	1.1 ± 4.1	1.5 ± 2.8	80.5 ± 2.5	1.1 ± 7.2	NG ^d^
10	Labiateae	*Achillea santolina *L.	100	80.8 ± 3.4	1.9 ± 2.8	NG ^d^	NG ^d^	90.1 ± 2.4	NG ^d^	1.9 ± 1.8
11	Labiateae	*Mentha piperita *L.	100	90.9 ± 8.7	NG ^d^	1.1 ± 9.4	NG ^d^	92.5 ± 8.4	NG ^d^	NG ^d^
12	Labiateae	*Origanum syriacum *L.	100	90.8 ± 2.9	NG ^d^	NG ^d^	1.1 ± 5.6	80.9 ± 4.5	NG ^d^	1.1 ± 1.9
13	Labiateae	*Phlomis brachydon *(Boiss.) Zohary	100	85.5 ± 5.6	1.1 ± 3.1	NG ^d^	NG ^d^	88.5 ± 7.3	NG ^d^	NG ^d^
14	Labiateae	*Teucrium polium *L.	100	75.5 ± 9.6	NG ^d^	NG ^d^	NG ^d^	80.5 ± 7.3	NG ^d^	NG ^d^
15	Leguminosae	*Anagyris foetida *L	100	102 ± 4.8	1.5 ± 4.1	NG ^d^	NG ^d^	100.5 ± 7.3	1.1 ± 3.2	NG ^d^
16	Leguminosae	*Trigonella foenum- graecum *L.	100	105.9 ± 4.1	NG ^d^	1.5 ± 6.6	NG ^d^	86.0 ± 6.4	1.1 ± 3.1	NG ^d^
17	Theaceae	*Thea sinensis *L.	100	100 ± 3.6	NG ^d^	NG ^d^	NG ^d^	92.3 ± 8.4	NG ^d^	NG ^d^
18	Malvaceae	*Hibiscus sabdariffa *L	100	100 ± 4.3	NG ^d^	NG ^d^	5.5 ± 2.2	70.9 ± 1.1	1.5 ± 4.5	NG^d^
19	Umbelliferae	*Lepidium sativum *L.	100	100 ± 1.6	NG ^d^	NG ^d^	1.1 ± 4.1	90.9 ± 2.3	1.1 ± 5.5	55.8 ± 3.2
20	Umbelliferae	*Pimpinella anisum *L.	100	90 ± 2.5	NG ^d^	NG ^d^	1.1 ± 9.4	75.5 ± 10.0	1.1 ± 1.8	NG ^d^

^a ^Amoxicillin (Amo), Chloramphenicol (Chl), Neomycin(Neo), Doxycycline (Doxy), Clarithromycin (Clarith), Cephalexin (Ceph) and Nalidixic acid (Nal).

^b^SE standard error

^c ^plant extracts concentrations was (200 μg/ml)

* Antibiotics concentrations were half the MICs (presented in Table 2)

^c ^Blank containing the solvent with the nutrient agar and the bacteria (allowed full growth of the microorganism (100%)

^d ^NG no detectable growth

Preliminary experiments were carried out to confirm that plant extracts at 200 μg/ml, the antibiotics at half their MICs, and the 0.5 ml of ethanol did not inhibit growth of the challenge inoculum. These experiments were also repeated every time the modification of antibiotic activity by plant extracts was studied.

## Results and Discussion

Plants used in this study are mentioned in Table 1. Some of these plants are edible used either as food or in the folk medicine and are considered safe. The rest of the plants are not commonly used by the laymen, but are used by herbalists in folk medicine [19-23].

The bacteria used in this study were resistant and standard strains of *E. coli*. In addition to being an essential component of the gut flora, *E. coli *is an etiologic agent for both hospital and community-acquired infections in humans [2,25,26]. As with other bacterial pathogens, this bacterium can develop single and multidrug resistance to several antimicrobial families; consequently, antimicrobial treatment of invasive *E. coli *infections can be challenging. The antibiotics used in this study were chosen to represent different groups of antibiotics. Their concentrations were chosen to be approximately half their MIC (Table 2) to guarantee that the effect produced is due to the combination and not to the effect of the antibiotic alone.

The effects of the plant extracts on the growth of the antibiotics against the resistant and standard strains of *E. coli *are shown in Tables 3 and 4. Samples of the plants without combinations allowed 100% growth of the inoculum at level of 200 μg ml^-1^. Methanolic extracts of the plant materials significantly enhanced the inhibitory effects of chloramphenicol, neomycin, doxycycline, cephalexin and nalidixic acid (Table 3 and 4) against both the standard strain and to a lesser extent the resistant strain of *E. coli*. The effects varied significantly according to the antibiotic and the *E. coli *strain. The efficacy of the combinations in enhancing the antibacterial activity was generally greater against the standard strain where for some combinations no growth was detected (e.g. combinations of chloramphenicol, neomycin, doxycycline, cephalexin and nalidixic acid with almost all plant material used). On the other hand, plant materials enhanced activity of these antibiotics to a slightly lesser extent against the resistant strain (Table 3).

Some of the plant materials used in the study like *Origanum syriacum *L. (Labiateae), Trigonella *foenum- graecum *L.(Leguminosae), *Euphorbia macroclada *(Euphorbiaceae) and *Hibiscus sabdariffa *(Malvaceae) did not enhance the activity of amoxicillin against both standard and resistant *E. coli *(Table 3 and 4). On the other hand combinations of amoxicillin with other plant materials used showed variable effect between standard and resistant strains. Plant material like *Anagyris foetida *(Leguminosae) and *Lepidium sativum *(Umbelliferae) reduced the activity of amoxicillin against the standard strain however; they enhanced the activity against resistant strains (Tables 3 &4).

Activity of cephalexin on the resistant strain was enhanced when used in combination with all plant materials (Table 3). The enhancement of the activity of cephalexin was more pronounced against the standard strain with all the plant materials used (Table 4). Of note is the fact that cephalexin is one of the first generation cephalosporins which do not normally have activity against *E. coli*. This might indicate that the plant material allowed better penetration of the drug through the outer layers to the cell wall, which is the target site for this antibiotic. This might also indicate that the plant material acts by another mechanism such as blocking the inhibitory effect of the enzymes.

Combinations of clarithromycin with three edible plants; *Gundelia tournefortii *L. (Compositae) *Eruca sativa *Mill. (Cruciferae), and *Origanum syriacum *L. (Labiateae), enhanced activity against the resistant *E. coli *strain (Table 3). However, combinations of this antibiotic with the other plant materials used did not enhance the inhibitory effect significantly against both standard and resistant strains.

The main mechanisms of resistance to antibiotics used in this study are active efflux and enzymatic inactivation [27]. Several studies have been performed to identify drugs interfering with these pumps, called resistance modifying agents [28]. Plant products, as ethanol extracts of *Mentha arvensis*, are known to affect the efflux system of an *E. coli *multiresistant to aminoglycosides, inhibiting these resistance mechanism [29]. This strategy is named "herbal shotgun" or "Synergistic multi-target effects" and refers to the use of herbals and drugs in a multi targeted approach, due to the fact that mono or multi-extract combinations affect not one but several targets, cooperating in an agonistic-synergistic way. This approach is not exclusive for extract combinations, but combinations between single natural products or extracts with chemosynthetic or antibiotics are possible too [30-32].

The observed variations in the activity of the combinations on the two strains indicate structural changes in standard and resistant strains. The observed variations in the effects when using different plants and plants belonging to the same families suggests different structure and mechanism of action for the active substance (s) in these plants (Table 3 and 4).

## Conclusion

On the basis of the evidence obtained from this study some general conclusions can be drawn regarding the effect of the plant material on the activity of antibiotics used. Different plants sometimes belong to the same family, have different effects on the activity of antibiotics. Neomycin, chloramphenicol, doxycycline and cephalexin can be given advantageously with almost all the plant materials mentioned earlier with few exceptions (e.g. with *Trigonella foenum-graecum*), however, clinical trials are required to support that. The activity of amoxicillin and clarithromycin were the least enhanced by the presence of the plant material against Gram negative bacteria. Doxycycline activity was the most significantly improved when combined with the plant material when tested against both bacterial strains. Nalidixic acid activity was improved significantly when combined with all plant materials and tested on standard strains. On the other hand, its activity on the resistant strain was slightly improved using the same combinations.

## Competing interests

The authors declare that they have no competing interests.

## Authors' contributions

RMD has carried out the microbiology experimental part such as inoculum preparation and antimicrobial evaluation. TAA collected the ethnomedicinal plants and performed the experimental part which involved plant material such as extraction. Both authors evaluated the results and corrected the manuscript for publication. Both authors read and approved the final manuscript.

## Pre-publication history

The pre-publication history for this paper can be accessed here:

http://www.biomedcentral.com/1472-6882/10/9/prepub
